# Accuracy of Diagnostic Tests for Detecting *Schistosoma mansoni* and *S. haematobium* in Sub-Saharan Africa: A Systematic Review and Meta-Analysis

**DOI:** 10.1155/2023/3769931

**Published:** 2023-08-16

**Authors:** Daniel Getacher Feleke, Yonas Alemu, Habtye Bisetegn, Habtu Debash

**Affiliations:** ^1^Department of Microbiology, Immunology and Parasitology, College of Health Sciences, Addis Ababa University, Addis Ababa, Ethiopia; ^2^Department of Medical Laboratory Science, College of Medicine and Health Sciences, Wollo University, Dessie, Ethiopia

## Abstract

**Introduction:**

Schistosomiasis is causing high morbidity and significant mortality in endemic areas. Kato-Katz stool examination and urine filtration techniques are the conventional methods for the detection of intestinal and urinary schistosomiasis. The most appropriate diagnostic tools for the detection of schistosomiasis especially in low-prevalence settings should be used. Therefore, this study is aimed at investigating the diagnostic accuracy of *S. mansoni* and *S. haematobium* diagnostic tools in sub-Saharan Africa.

**Methods:**

Electronic databases such as PubMed, PubMed Central/Medline, HINARI, Scopus, EMBASE, Science Direct, Google Scholar, and Cochrane Library were reviewed. The pooled estimates and heterogeneity were determined using Midas in Stata 14.0. The diagnostic accuracy of index tests was compared using the hierarchical summary of the receiver operating characteristic (HSROC) curve in Stata 14.0.

**Results:**

Twenty-four studies consisting of 12,370 individuals were tested to evaluate the accuracy of antigen, antibody, and molecular test methods for the detection of *S. mansoni* and *S. haematobium*. The pooled estimate of sensitivity and specificity of CCA was 88% (95% CI: 83-92) and 72 (95% CI: 62-80), respectively, when it is compared with parasitological stool examination for *S. mansoni* detection. On the other hand, ELISA showed a pooled estimate of sensitivity and specificity of 95% (95% CI: 93-96) and 35% (95% CI: 21-52), respectively, for the examination of *S. mansoni* using stool examination as a reference test. With regard to *S. haematobium*, the pooled estimate of sensitivity and specificity of polymerase chain reaction was 97% (95% CI: 78-100) and 94% (95% CI: 74-99), respectively. Moreover, the sensitivity and specificity of urine CCA vary between 41-80% and 55-91%, respectively, compared to urine microscopy.

**Conclusion:**

The effort of schistosomiasis elimination requires accurate case identification especially in low-intensity infections. This study showed that CCA had the highest sensitivity and moderate specificity for the diagnosis of *S. mansoni*. Similarly, the sensitivity of ELISA was excellent, but its specificity was low. The diagnostic accuracy of PCR for the detection of *S. haematobium* was excellent compared to urine microscopic examination.

## 1. Introduction

Schistosomiasis is one of the most prevalent neglected tropical diseases in the rural areas of developing countries [1, 2]. An estimated 200 million people, 111 million school-aged children and 95 million adults, are at risk of infection [3, 4]. In developing countries, schistosomiasis is responsible for the deaths of 300,000 people annually [5]. In human, *Schistosoma haematobium*, *S. mansoni*, *S. japonicum*, *S. mekongi*, and *S. intercalatum* are the main species responsible for the majority of schistosomiasis cases [6]. Among these species, *S. mansoni* and *S. haematobium* are the two most prevalent species in sub-Saharan Africa [3, 4]. Schistosomiasis causes high morbidity and significant mortality in endemic areas [7].

In developing countries, children aged 5–17 years are at risk of infection due to their frequent contact with water [4, 6]. The risk factors associated with schistosomiasis include poor sanitation, bathing and swimming in dams and rivers, uses of unprotected water sources, and lack of awareness about the prevention and controls of schistosomiasis [2, 4]. Schistosomiasis transmission occurs when infected individuals contaminate freshwater sources with their excreta containing parasite eggs, which later hatch in water [8]. In freshwater, these eggs form miracidia, which hatch and infect snails. Then, the miracidium develops into sporocysts in snail and produces cercaria [9]. Humans are usually infected when cercaria penetrates the skin during contact with contaminated freshwater [8, 9].

The World Health Organization utilizes traditional techniques such as the Kato-Katz stool examination and urine filtration methods to detect the presence of schistosomiasis eggs in both stool and urine samples. These methods involve microscopic examination and are still widely employed for the diagnosis of intestinal and urinary forms of schistosomiasis [8, 10].

However, Kato-Katz and urine filtration techniques are known to have low sensitivity for the detection of light-intensity infections. In recent years, serological techniques for the detection of antibodies against Schistosoma antigens were developed. The techniques include indirect immunofluorescent-antibody tests (IFATs), indirect hemagglutination assays (IHAs), and enzyme-linked immunosorbent assays (ELISAs) using different antigens [11]. Moreover, there are several different nucleic acid-based methods for the detection of helminth infections such as PCR, multiplex PCR, qPCR, and LAMP. These methods offer advantages such as precision, timeliness, and sensitivity of parasite detection [12]. Detection cell free DNA (cfDNA) in host blood may also be a promising diagnostic marker for the detection of Schistosoma parasite [13]. The performance of diagnostic tools of *S. mansoni* and *S. haematobium* should be investigated to choose the most appropriate diagnostic tools for the detection of schistosomiasis especially in low-prevalence settings. The diagnostic test methods used for the diagnosis schistosomiasis must have sufficient level of accuracy beside other test method selection criteria. Therefore, this systematic review and meta-analysis is aimed at investigating the diagnostic accuracy of antigen, antibody, and molecular tests of published studies for *S. mansoni* and *S. haematobium* detection in sub-Saharan Africa.

## 2. Materials and Methods

### 2.1. Study Protocol, Registration, and Search Strategies

This systematic review was registered on PROSPERO (CRD42021274085) and reported using the preferred reporting items for systematic review and meta-analysis (PRISMA) guidelines (supplementary file 1) [11]. Studies that assessed the diagnostic accuracy of antigen, antibody, and molecular tests used for the detection of *S. mansoni* and *S. haematobium* using parasitological and urine examination as reference method in sub-Saharan African countries were reviewed. The diagnostic methods include ELISA, CCA, PCR, SmCTF antibody test, and Sm-IHA for the detection of S. mansoni and *S. haematobium* using parasitological examination as a reference test method. Only studies in English language were included in this systematic review and meta-analysis.

In this study cross-sectional, cohort and case controls were reviewed. However, case-control studies with healthy controls and those with qualitative outcomes were excluded. Moreover, case studies, conference proceedings, and commentaries were excluded.

The electronic databases searched were PubMed, PubMed Central/Medline, HINARI, Scopus, EMBASE, Science Direct, Google Scholar, Cochrane Library, and proceedings of health professional associations and theses of universities (supplementary file 2). The search was conducted from July to August 2021. The key words used to retrieve all relevant articles includes “Schistosomiasis”, Snail fever, bilharzia “*S. mansoni*”, “*S. haematobium*”, “serologic tests”, “Antigen tests”, “Antibody tests” “molecular tests”, “microscopy” “diagnostic”, “diagnosis”, and “Sub-Saharan Africa.” These key words were used in combination using Boolean operators. Moreover, reference lists of the included studies were searched.

### 2.2. Selection of Studies

All retrieved studies were screened by the authors (DGF, YA, HB, and HD) independently based on their title and abstract. Duplicated studies and those studies without appropriate reference test method were excluded. Disagreements on the eligibility of studies were solved after detailed discussions with all authors. Abstracts without full text were excluded after requesting authors for full text.

### 2.3. Data Extraction and Quality Assessment

Data were extracted independently by the authors (DGF, YA, HB, and HD) using data extraction form developed based on the objective of this study. The characteristics of the selected studies that include authors, publication year, study country, number of participants, study population, number of cases, study design, reference test, index tests, and data for 2 × 2 tables (true positive, false positive, false negative, and true negative) were collected. The extracted data were cross-checked, and disagreements were solved by discussion with all authors. Quality assessment of diagnostic accuracy studies (QUADAS-2) tool was used to assess the methodological quality of the included studies by all authors independently (DGF, YA, HB, and HD). The risk of bias summary and graph were generated using Review Manager 5 (RevMan version 5.4.1). Quality assessment of diagnostic accuracy studies (QUADAS-2) assessment had four domains. These domains are patient selection, index test, reference standard, and flow and timing.

Multiple 2 × 2 tables were extracted from a single study if it compared more than one index tests with a reference test standard. Moreover, each test comparisons of a single study were independently assessed for methodological quality.

### 2.4. Statistical Analysis

The estimates (with 95% confidence interval) of sensitivity, specificity, positive likelihood ratio, negative likelihood ratio, and diagnostic odds ratio for each index test were plotted in forest plots using Review Manager 5.4.1. The summary estimates and heterogeneity of the included studies were determined using Midas in Stata 14.0. Diagnostic odds ratio (DOR) was used to determine the overall diagnostic accuracy, when making comparisons between index tests. Comparison of diagnostic accuracy among different index tests was done using hierarchical summary of receiver operating characteristic (HSROC) curve using HSROC model in Stata 14.0.

## 3. Results

### 3.1. Search Results and Eligible Studies

**Figure 1:**
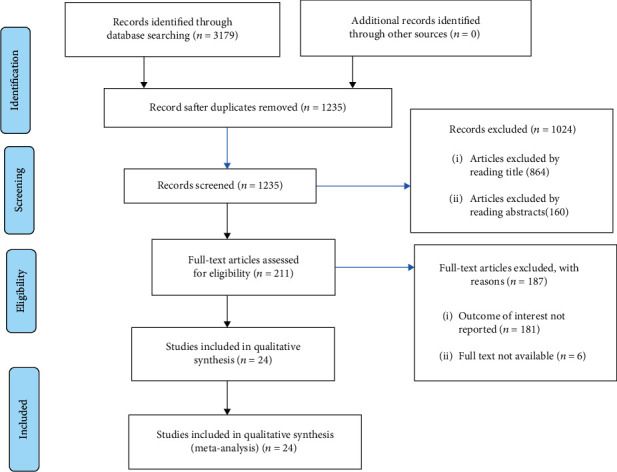
Flowchart of study selection.

The systematic literature search identified 3,179 records from different sources. In this study, 24 studies were eligible to be included. The remaining studies were excluded due to reasons such as duplicate records, studies that were not related to the objective, incomplete data for extracting 2 × 2 tables, studies that were not comparing diagnostic tests, and studies that used reference tests other than parasitological test (Figure 1).

### 3.2. Study Characteristics

Twenty-four studies consists of 12,370 individuals that were tested to evaluate the accuracy antigen, antibody, and molecular tests using stool and urine examinations as reference test methods. Out of the 24 included studies, 18 studies (9,536 individuals) reported evaluation of test methods for *S. mansoni* and the remaining 6 studies (2,834 individuals) evaluated test methods for *S. haematobium*. Majority of the studies (21/24) were conducted among children. Most of the included studies were cross-sectional studies (21/24). The remaining studies were longitudinal survey and case control.

The diagnostic methods compared in this study includes circulating cathodic antigen (CCA), enzyme-linked immunosorbent assay (ELISA), polymerase chain reaction (PCR), S. mansoni cercarial transformation fluid (SmCTF antibody test), and *S. mansoni* indirect hemagglutination assay (Sm-IHA) using stool and urine examinations as a reference test method. Enzyme-linked immunosorbent assay detects different antigens of Schistosoma parasite. The SWAP-ELISA targets soluble S. mansoni adult worm antigens, the antigen preparation of SEA-ELISA is soluble S. mansoni egg antigens, and the CEF6-ELISA used cationic exchange fraction of S. mansoni egg antigen. Circulating cathodic antigen (CCA) and circulating anodic antigen (CAA), both produced by the gut epithelium of living young and adult worms, were used to detect Schistosoma parasite. Schistosoma mansoni cercarial transformation fluid (SmCTF) is a method for detecting antischistosomal antibodies in human blood. Circulating cathodic antigen (CCA) (15,118 tests) and ELISA (1,885 tests) test methods were evaluated for the detection of S. mansoni. There were only two studies each that compare PCR (383 tests) and SmCTF antibody test (199 tests) with parasitological stool examination for the detection of S. mansoni. There was also one study that evaluated Sm-IHA (205 tests) against stool examination for S. mansoni detection. These studies were not pooled because of the small number of studies. However, they were reported and discussed in this study (Table 1). Regarding S. haematobium, there were studies that compare CCA (3,454 tests), PCR (2,224 tests), and antibody tests (307 tests) with urine examination (Table 2).

### 3.3. Data Quality Assessment and Heterogeneity of the Included Studies

**Figure 2:**
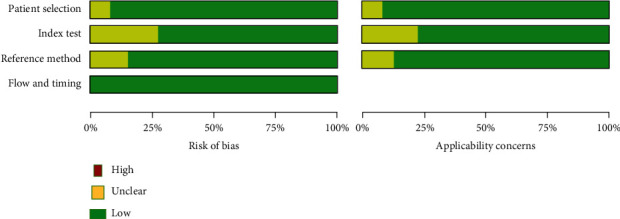
Risk of bias graph of studies included in the meta-analysis.

**Figure 3:**
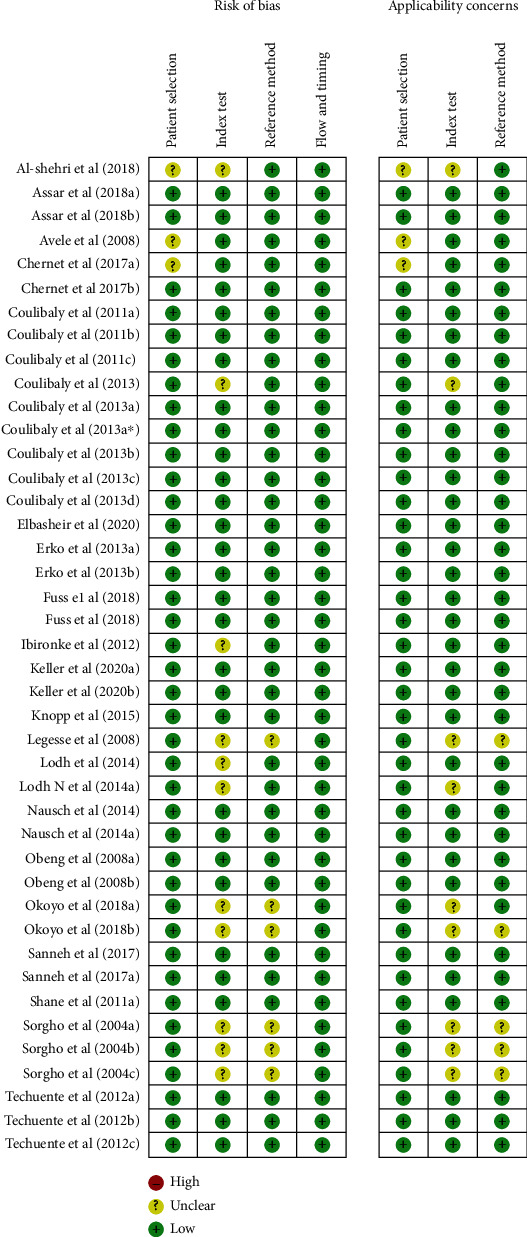
Risk of bias and applicability concern summary for each domain of the included studies.

Quality assessment of diagnostic accuracy studies (QUADAS-2) assessment showed that there was no high risk of bias in all the four domains. The risk of bias in patient selection domain was considered low in almost 95% of the diagnostic studies. The quality of verification with a flow and timing was excellent in all of the studies. There were few studies with unclear risk of bias especially in the index test and reference standard domains (Figures 2 and 3).

There was high heterogeneity between studies included for comparing CCA (*Q* = 888.489, *I*^2^ = 100%, *P* < 0.001) and ELISA (*Q* = 19.360, *I*^2^ = 90%, *P* < 0.001) with parasitological stool examination for the diagnosis of *S. mansoni*. With regard to *S. haematobium*, studies that compare PCR (*Q* = 22.964, *I*^2^ = 91%, *P* < 0.001) and CCA (*Q* = 8.545, *I*^2^ = 77, *P* = 0.007) with parasitological urine examination had considerable heterogeneity. The source of heterogeneity was explored through the threshold effect analysis. The results suggested that there was no threshold effect between studies that compare CCA with urine examination (*P* = 0.27) for the detection of *S. haematobium*. In contrast to this, there was threshold effect between studies that compare PCR (*P* = 0.02) with urine examination. On the other hand, the source of heterogeneity between studies that compare ELISA (*P* = 1.00) and CCA (*P* = 0.36) with stool examination for the detection of *S. mansoni* was not due to threshold effect.

### 3.4. CCA with Parasitological Stool Examination as Reference Test for the Detection of *S. mansoni*

**Figure 4:**
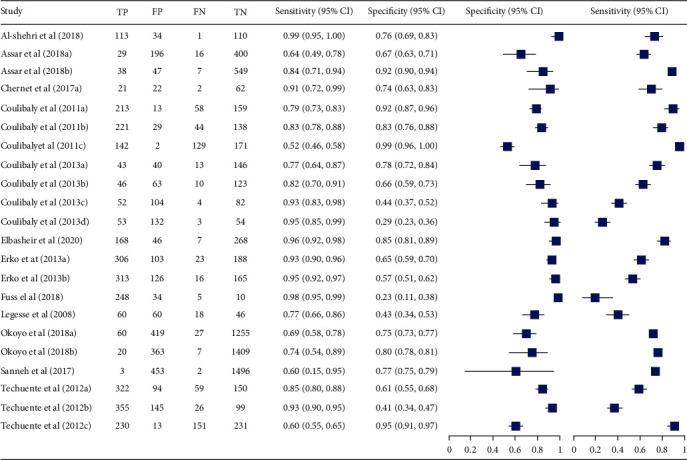
Forest plots of sensitivity and specificity of CCA compared with parasitological stool examination as reference test method.

**Figure 5:**
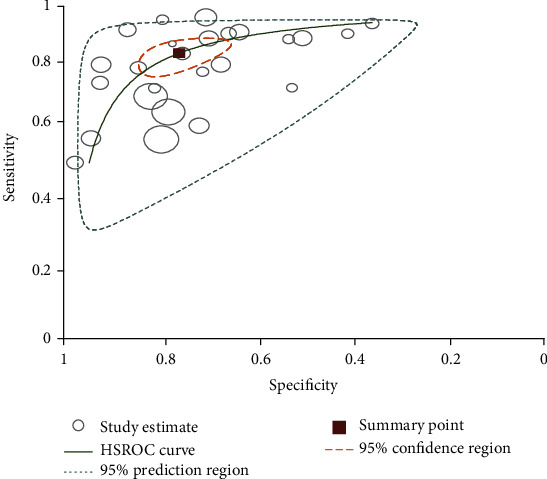
Summary receiver operating characteristic plot of sensitivity and specificity of CCA with parasitological stool examination as a reference test.

Fourteen studies (15,118 tests) were included to compare circulating cathodic antigen (CCA) with parasitological stool examination (Kato-Katz test, formalin-ethyl ether sedimentation concentration technique (FECT), and cellophane) [14–24, 28]. Majority of the studies used Kato-Katz technique as a reference test method. The sensitivity and specificity of CCA of these studies varies between 52%-100% and 23%-99%, respectively. The summary estimate of sensitivity and specificity of CCA was 88% (95% CI: 83-92) and 72 (95% CI: 62-80), respectively. Moreover, it had also diagnostic odds ratio of 19 (95% CI: 13-30), positive likelihood ratio (LR+) of 3.2 (2.4-4.3), and negative likelihood ratio (LR-) of 0.16 (0.12-0.23). The area under the curve (AUC) was 0.89 (95% CI: 0.86-0.91) (Figures 4 and 5).

### 3.5. ELISA, PCR, and SmCTF Antibody Test with Stool Examination as Reference Test for the Detection of *S. mansoni*

Three studies that compare ELISA with parasitological stool examination tested 1,885 individuals [24, 27, 29]. The summary estimate of sensitivity and specificity of ELISA was 95% (95% CI: 93-96) and 35% (95% CI: 21-52), respectively. It had diagnostic odds ratio of 9 (95% CI: 5-18), positive likelihood ratio of 1.4 (95% CI: 1.1-1.8), and negative likelihood ratio of 0.15 (95% CI: 0.10-0.24), respectively. The summary receiver operating characteristic plot showed that the AUC of ELISA was 93% (95% CI: 91-95).

On the other hand, there were only two studies that compare SmCTF antibody test with stool examination. The sensitivity of these studies was higher than their specificity.

**Figure 6:**
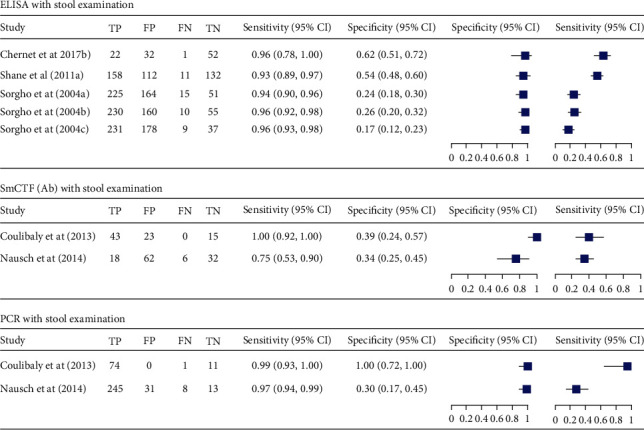
Forest plots of sensitivity and specificity of ELISA, PCR, and SmCTF antibody test using stool examination as reference test method.

**Figure 7:**
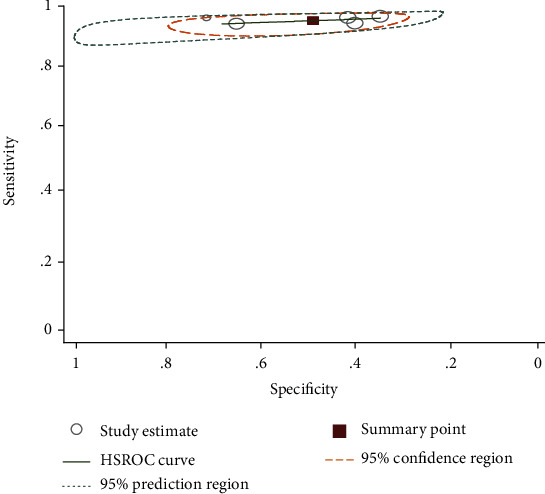
Summary receiver operating characteristic plot of sensitivity and specificity of ELISA with stool examination as a reference test method.

Another two studies that evaluated PCR against stool examination showed a sensitivity of above 95% and specificity of 100%. One of these studies showed lower specificity 30% (Figures 6 and 7). Furthermore, there was one study that evaluated Sm-IHA against stool examination, and it showed a sensitivity and specificity of 85% and 34%, respectively.

### 3.6. PCR, CCA, and SmCTF Antibody Test with Parasitological Urine Examination as Reference for the Detection of *S. haematobium*

**Figure 8:**
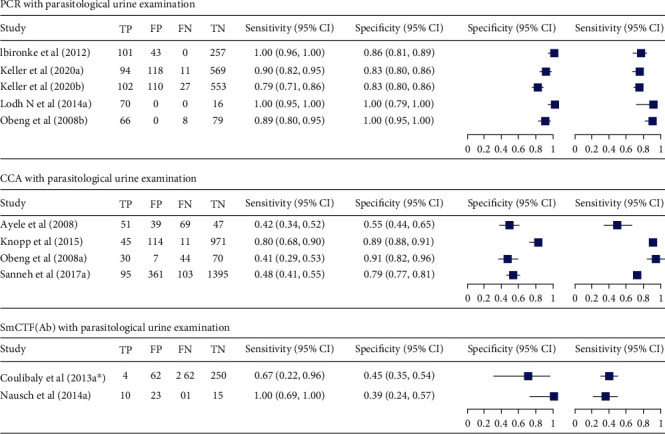
Forest plots of sensitivity and specificity of ELISA, SmCTF (Ab), and PCR using stool examination as reference test method.

Studies comparing PCR with parasitological urine examination tested 2,224 individuals [25, 31, 32, 36]. The sensitivity and specificity of these studies vary between 79-100% and 83-100%, respectively. The observed sensitivity and specificity of urine CCA compared with parasitological urine examination were 41-80% and 55-91%, respectively. The area under the curve (AUC) of CCA was 0.71. There were two studies [20, 36] that evaluated SmCTF antibody test against parasitological urine examination. They showed higher sensitivity and lower specificity (Figure 8).

**Figure 9:**
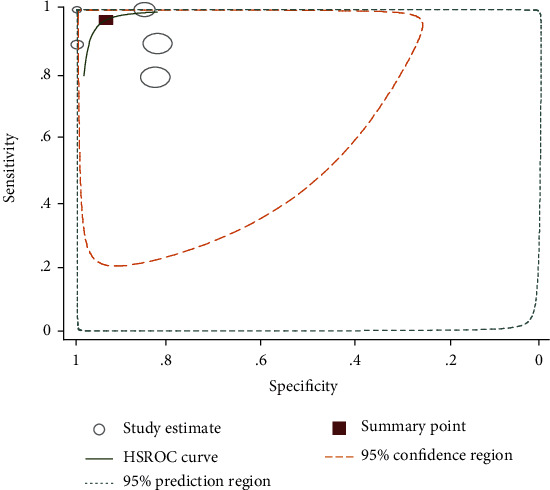
Summary receiver operating characteristic plot of sensitivity and specificity of PCR with urine examination as a reference test method.

There was also one study that evaluated monoclonal antibody dipstick-ELISA, and it showed a sensitivity of 98.8% and specificity of 53.6% [29]. The pooled estimate of sensitivity and specificity of PCR was 97% (95% CI: 78-100) and 94% (95% CI: 74-99), respectively. It also showed an excellent accuracy with AUC of 0.96 (Figure 9).

The diagnostic odds ratio of urine CCA and PCR was 478 (95% CI: 27-8527) and 5 (1-17), respectively (Table 3).

**Figure 10:**
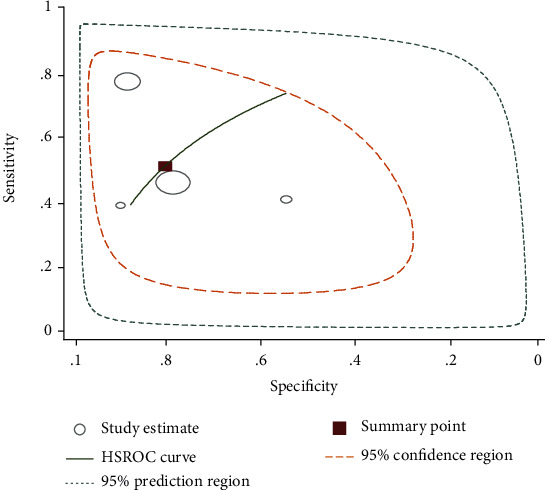
Summary receiver operating characteristic plot of sensitivity and specificity of CCA with urine examination as a reference test method.

The summary estimate of sensitivity and specificity of CCA was 53% (95% CI: 37-69) and 81% (95% CI: 66-90) (Figure 10).

## 4. Discussion

Schistosomiasis is one of the most prevalent neglected tropical diseases (NTDs) in the rural areas of developing countries [1–3]. *Schistosoma mansoni* and *S. haematobium* are the two most prevalent species that affect nearly 192 million people in more than 40 countries of the sub-Saharan region [7].

In this systematic review and meta-analysis, parasitological examination of stool and urine was used as reference test methods to compare antigen, antibody, and molecular tests for the detection of *S. mansoni* and *S. haematobium*, respectively. Of the 24 eligible and included studies, 18 were compared using ELISA, CCA, PCR, SmCTF antibody test, and Sm-IHA for the detection of *S. mansoni* and *S. haematobium* using parasitological examination as a reference test method.

Statistical heterogeneity was tested using the *I*^2^ statistic that measures the variation across studies due to interstudy heterogeneity. There was significant heterogeneity among the included studies in this meta-analysis. This heterogeneity is expected to be related to the method of test reading, the number of slide and stool/urine samples examined for reference and index tests, and the intensity of infection. The diagnostic accuracy of CCA and ELISA based on the summary estimates for the detection of *S. mansoni* showed a sensitivity of 88% and 95%, respectively, using stool examinations as a reference test. However, the specificity was 72% and 35%, respectively. The area under the curve (AUC) of CCA was 0.89 (95% CI: 0.86-0.91) that indicates that CCA has very good diagnostic accuracy for diagnosing *S. mansoni* infection.

The ELISA test for *S. mansoni* detected a large proportion of infections detected by stool examination (sensitivity: 95%, 95% CI: 93-96). It also identified a large proportion of individuals negative by stool examinations as positive (specificity: 35%, 95% CI: 21-52). This might be due to the low sensitivity of Kato-Katz technique for the detection of light-intensity infections. The discrepancy of results between Kato-Katz technique and index tests taking into account the real result of an individual could be improved by collecting all the clinical and epidemiological data and also performing multiple microscopic tests on the same sample or on another sample. This is helpful to not neglect the real specificity of index tests due to the low sensitivity of standard test method.

The ELISA technique used in this study was based on the detection of *S. mansoni* antibody that cannot differentiate current and past infections. Moreover, the included studies used different antigens for detecting antibodies using ELISA that may have different sensitivity [26]. One of the studies that used soluble egg antigen (SEA) for detection of specific antibody by ELISA method showed highest sensitivity (96%) and moderate specificity (62%) (Figure 5). Generally, in the present study, CCA and ELISA showed high accuracy for the detection of *S. mansoni* using stool examination as a reference test method. As the number of studies was few, pooled estimates for PCR and SmCTF antibody test for the detection of *S. mansoni* were not generated. However, the diagnostic accuracy of PCR obtained from two studies showed highest sensitivity (above 95%) and specificity (100%) compared to stool examination. One of these studies had low specificity [25]. This might be due to variations of test accuracy caused by using different sample types for *S. mansoni* diagnosis.

With regard to *S. haematobium*, there were sufficient studies that compare PCR and CCA with parasitological urine examination. The pooled estimate of sensitivity and specificity of PCR was 97% (95% CI: 78-100) and 94% (95% CI: 74-99), respectively. PCR had 478 times higher odds of obtaining positive result in diseased individuals than in the nondiseased. It had also an excellent diagnostic accuracy with AUC of 0.96. On the other hand, the summary estimate of sensitivity and specificity of urine CCA was 53% (37-69%) and 81 (66-90%), respectively. Meta-analysis was not conducted for SmCTF antibody test for the detection of *S. haematobium* due to insufficient number of the included studies. However, the available two studies had a sensitivity of 67% and 100% and a specificity of 39% and 45%.

Diagnosis of schistosomiasis requires sensitive diagnostic tools especially in low transmission areas of sub-Saharan Africa. The present study was challenged to come up with uniform comparison of index tests with the reference standard test. In this study, the included studies used different stool examination techniques (Kato-Katz, FECT, and sedimentation technique) and variety number of slide and stool samples examined per individual. These factors definitely contributed to variation of diagnostic efficacies with a wide range of estimated performance. With regard to the index tests, the pooling of ELISA detecting antibody with different antigens and CCA of different types, number, and result interpretation (considering trace positive or negative) to detect *S. mansoni* and *S. haematobium* may cause variation of diagnostic accuracy of the test methods. This study also pooled different types of PCR (RTPCR, qPCR, and conventional PCR) with several types of specimens. Therefore, the diagnostic performance of PCR is known to differ by the sample type used [37, 38], which may also have had an effect on the diagnostic accuracy of PCR in this study.

## 5. Conclusion

The effort of schistosomiasis elimination requires accurate identification of infections using sensitive diagnostic tests especially in low-intensity infections. This systematic review and meta-analysis assessed the performance of antigen, antibody, and molecular tests for detecting *S. mansoni* and *S. haematobium* that was challenged by a limited number of published studies that meet selection criteria in some of the test categories. CCA tests evaluated for *S. mansoni* infection using stool examination as a reference standard showed the highest sensitivity and moderate specificity. The sensitivity of ELISA was excellent but with low specificity for the detection of *S. mansoni* infection. The diagnostic accuracy of PCR for the detection of *S. haematobium* infection was found to be excellent using urine examination as to reference test method.

## Figures and Tables

**Table 1:** Characteristics of the studies included in the systematic review and meta-analysis of accuracy of diagnostic tests for *S. mansoni*.

S. no.	Author (year)	Study population	Country	Sample size	Index tests	Reference test
1	Legesse et al. (2008) [14]	School children	Ethiopia	184	CCA	Kato-Katz and formol ether
2	Okoyo et al. (2018) [15]	School children	Kenya	1,761	POC-CCA	Kato-Katz smear
3	Tchuem Tchuenté et al. (2012) [16]	School children	Cameroon	625	One CCA, three CCA, one CCA-L	9 × Kato − Katz
4	Coulibaly et al. (2011) [17]	School children	Côte d'Ivoire	446	One CCA-A, three CCA-A, one CCA-B	9 × Kato − Katz
5	Coulibaly et al. (2013) [18]	Preschool children	Côte d'Ivoire	242	Single and duplicate POC-CCA	4 × Kato − Katz
6	Erko et al. (2013) [19]	School children	Ethiopia	620	Single and triple urine CCA	6 × Kato − Katz
7	Sanneh et al. (2017) [20]	School children	Gambia	1,954	POC-CCA	Kato-Katz smear
8	Elbasheir et al. (2020) [21]	School children	Sudan	489	Urine CCA	Kato-Katz smear
9	Assaré et al. (2018) [22]	Children 9-12	Côte d'Ivoire	641	POC-CCA with and without trace	Kato-Katz smear
10	Fuss et al. (2018) [23]	School children	Tanzania	297	CCA, PCR	Kato-Katz smear
11	Chernet et al. (2017) [24]	Asymptomatic adult	Eritrea	107	POC-CCA, ELISA	Sedimentation
12	Lodh et al. (2014) [25]	Children	Ghana	86	PCR	Kato-Katz smear
13	Nausch et al. (2014) [26]	School children	Zimbabwe	81	SmCTF (Ab)	Kato-Katz, urine filtration
14	Coulibaly et al. (2013) [18]	Preschool children	Côte d'Ivoire	118	SmCTF (Ab)	Kato-Katz, urine filtration
15	Sorgho et al. (2005) [27]	General population	Burkina Faso	455	SWAP-ELISA, SEA-ELISA, CEF6-ELISA, Sm-IHA	Kato-Katz smear
16	Al-Shehri et al. (2018) [28]	School children	Uganda	258	Urine CCA	Kato-Katz smear
17	Shane et al. (2011) [29]	Children	Kenya	413	Carbon CCA, cassette CCA	Kato-Katz smear
18	Mewamba et al. (2021) [30]	School children	Cameroon	759	POC-CCA	Kato-Katz smear

**Table 2:** Characteristics of the studies included in the systematic review and meta-analysis of accuracy of diagnostic tests for *S. haematobium*.

S. no.	Author (year)	Study population	Country	Sample size	Index tests	Reference test
1	Ibironke et al. (2012) [31]	Adult population	Nigeria	401	PCR	Urine filtration (microscopy)
2	Keller et al. (2018) [32]	School children and adult population	Zanzibar	792	qPCR	Urine filtration (microscopy)
3	Bosompem et al. (2004) [33]	School children	Ghana	141	Monoclonal (Ab)	Urine microscopy
4	Knopp et al. (2015) [34]	Children	Zanzibar	1,141	Urine CCA	Urine filtration (microscopy)
5	Ayele et al. (2008) [35]	School children	Ethiopia	206	CCA	Urine filtration (microscopy)
6	Obeng et al. (2008) [36]	School children	Ghana	153	RT-PCR, carbon CCA	Urine filtration (microscopy)

**Table 3:** Summary estimates of diagnostic accuracy for *S. haematobium* using urine examination as a reference method.

	PCR		CCA	
	Estimate (%)	95% CI	Estimate%	95% CI
Sensitivity	97	78-100	53	37-69
Specificity	94	74-99	81	66-90
Diagnostic odds ratio	478	27-8527	5	1-17
Positive likelihood ratio	16.1	3.2-80.6	2.8	1.2-6.5
Negative likelihood ratio	0.03	0.00-0.28	0.58	0.37-0.90

## Data Availability

The authors confirm that all data underlying the findings are fully available without restriction. All relevant data are within the manuscript.

## Supplementary Materials

Supplementary 1 PRISMA checklist for reporting meta-analysis.

Supplementary 2 Full search strategy of an electronic database (PubMed).
