# Supplementary material for: Accuracy of Diagnostic Tests for Detecting Schistosoma mansoni and S. haematobium in Sub-Saharan Africa: A Systematic Review and Meta-Analysis
Source: Biomed Res Int. 2023 Aug 16;2023:3769931. doi: 10.1155/2023/3769931 (PMC10447154; doi:10.1155/2023/3769931)
Supplement: Supplementary 2 — Full search strategy of an electronic database (PubMed). [file 3769931.f2.docx]

**Full electronic search strategy on PubMed**

(schistosoma [MeSH] OR schistosomiasis [MeSH] OR schistosom* [tw] OR "schistoma infection" [tw] OR "schistosom* infection" [tw] OR bilharzia* [tw] OR bilharzio* [tw] OR "snail fever" [tw] OR "katayama fever" [tw] AND "schistosoma mansoni" [MeSH] OR "schistosoma mansoni" [tw] OR "s mansoni" [tw] OR "S. mansoni" [tw] OR "schistosoma haematobium" [tw] OR "s haematobium" [tw] OR "S. haematobium" [tw]) AND (serology [MeSH] OR serology [tw] OR "serologic* test*" [tw] OR "antigen* test*" [tw] OR ((antigens [MeSH] OR antigen [tw]) AND tests [tw]) OR microscopy [MeSH] OR microscopy [tw] OR "molecular test*" [tw] OR "laboratory test*" [tw] OR "rapid diagnostic test*" [tw] OR ((antibody [MeSH] OR antibody [tw]) AND test* [tw]) OR "antibody test*" [tw]) AND Sub-Saharan Africa
